# Epidemic Meningococcal Meningitis, Cameroon

**DOI:** 10.3201/eid1711.110468

**Published:** 2011-11

**Authors:** D. Massenet, D. Vohod, H. Hamadicko, D.A. Caugant

**Affiliations:** Centre Pasteur du Cameroun, Annexe de Garoua, Cameroon (D. Massenet); Hôpital Régional de Ngaoundéré, Ngaoundéré, Cameroon (D. Vohod); Délégation Régionale de la Santé Publique, Ngaoundéré (H. Hamadicko); Norwegian Institute of Public Health, Oslo, Norway (D.A. Caugant)

**Keywords:** Neisseria meningitidis, bacteria, meningococcal meningitis, epidemic, ST7, Cameroon

**To the Editor:** In 2010, the city of Ngaoundéré in Cameroon experienced its first reported epidemic of meningococcal meningitis. Ngaoundéré, with an estimated population of 180,000, is the main city in the Adamaoua region in northern Cameroon. The 2 northernmost regions of Cameroon, North and Far North, are considered to belong to the African meningitis belt (*1*) and are periodically affected by meningococcal meningitis outbreaks. However, the Adamaoua region had been spared because of its altitude, latitude, and low population density in comparison with the North and Far North regions. Fewer than 10 sporadic cases have been reported in the Adamaoua region every year.

During February–April 2010, a total of 126 cases of meningitis (70 cases/100,000 inhabitants) were reported in the Adamaoua region. Of the 126 cases, 34 were confirmed by identification of *Neisseria meningitidis* serogroup A in cerebrospinal fluid (CSF) samples, 46 cases were apparent meningitis in which the patients had turbid CSF, and 46 were clinical cases diagnosed in an epidemic context. The male:female ratio of the patients was 2.7:1. The mean age of patients was 19 years, and median was 17 years.

CSF specimens from 34 patients were sent to the Centre Pasteur du Cameroun in Garoua for testing. Laboratory procedures included assessing CSF turbidity, Gram staining, searching for soluble capsular antigens by using the Pastorex latex agglutination kit (Bio-Rad, Hercules, CA, USA), and testing by the dipstick rapid diagnostic test for *N. meningitidis* serogroups A, C, W135, and Y (provided by the Centre de Recherche Médicale et Sanitaire, Niamey, Niger).

All 34 specimens were positive for serogroup A by agglutination, rapid test, or both. CSF specimens were cultured on blood agar and chocolate agar supplemented with PolyViteX (bioMérieux, Marcy-l’Etoile, France) and incubated at 37°C in an atmosphere of 5% CO_2_. Susceptibility to antimicrobial drugs was tested according to the recommendations of the Antibiogram Committee of the French Society for Microbiology (www.sfm.asso.fr). An isolate of *N. meningitidis* was sent to the World Health Organization Collaborating Centre for Reference and Research on Meningococci in Oslo, Norway, for molecular analyses, as described (www.neisseria.org). The result was that the isolate, a *N. meningitidis* serogroup A clone of sequence type 7, was susceptible to β-lactams and chloramphenicol but resistant to trimethoprim/sulfamethoxazole.

This epidemic occurred in an area where the mean annual rainfall for the past 30 years was 1,460 mm (Agency for Aerial Navigation Safety in Africa and Madagascar, unpub. data). This value should exclude Ngaoundéré from the African meningitis belt, for which the southern limit of annual rainfall was classically considered to be the 1,100-mm isohyet (Figure).

**Figure:**
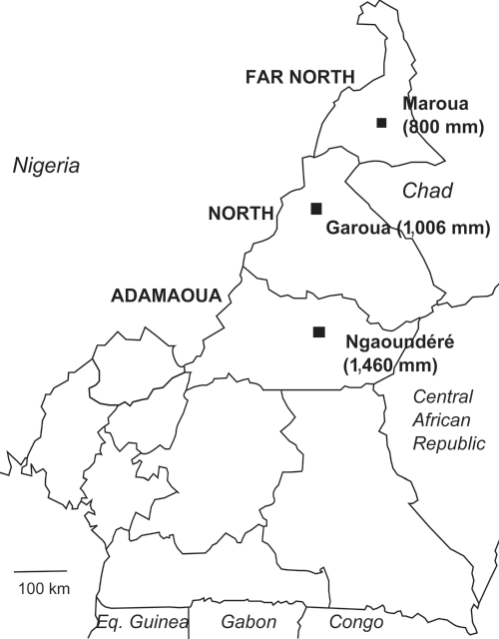
Northern regions of Cameroon with mean annual rainfall. Maroua is at the 800 mm isohyet line, Garoua at 1,006 mm, and Ngaoundéré at 1,460 mm. Estimate for Maroua is by the Agency for Aerial Navigation Safety in Africa and Madagascar; recorded rainfall for Garoua and Ngaoundéré are by the Agency for Aerial Navigation Safety in Africa and Madagascar. Eq. Guinea, Equatorial Guinea.

This epidemic at the border of the African meningitis belt raises the question of the belt limitation and its potential expansion southward. These topics should be addressed through active and standardized surveillance in countries such as Cameroon, which are not entirely included in the belt (*2*,*3*).

This meningitis epidemic has 2 other noteworthy characteristics. First, 80 (63%) of 126 suspected cases had a lumbar puncture performed at the Ngaoundéré Regional Hospital or at the Norwegian hospital. With the help of the laboratory, an increasing number of cases of meningitis in Cameroon are confirmed cases (*4*). Second, the etiologic agent was serogroup A meningococcus, a serogroup that had not been identified in north Cameroon since 2006 (*5*) but that had been isolated previously (*6*) and in south Cameroon (*7*).

## References

[1] Lapeyssonnie L. Cerebrospinal meningitis in Africa [in French]. Bull World Health Organ. 1963;28(Suppl):1–114. 14259333 PMC2554630

[2] Molesworth AM, Thomson MC, Connor SJ, Cresswell MP, Morse AP, Shears P, Where is the meningitis belt? Defining an area at risk of epidemic meningitis in Africa. Trans R Soc Trop Med Hyg. 2002;96:242–9. 10.1016/S0035-9203(02)90089-1 12174770

[3] Cuevas LE, Jeanne I, Molesworth A, Bell M, Savory EC, Connor SJ, Risk mapping and early warning systems for the control of meningitis in Africa. Vaccine. 2007;25(Suppl 1):A12–7. 10.1016/j.vaccine.2007.04.034 17517453

[4] Massenet D, Inrombe J. Impact of the biological monitoring of cerebrospinal meningitis on the notification of cases in North Cameroon [in French]. Rev Epidemiol Sante Publique. 2009;57:451–3. 10.1016/j.respe.2009.08.010 19896785

[5] Massenet D, Inrombe J, Mevoula DE, Nicolas P. Serogroup W135 meningococcal meningitis, northern Cameroon, 2007–2008. Emerg Infect Dis. 2009;15:340–2. 10.3201/eid1502.080988 19193290 PMC2662656

[6] Riou JY, Djibo S, Sangare L, Lombart JP, Fagot P, Chippaux JP, A predictable comeback: the second pandemic of infections caused by *Neisseria meningitidis* serogroup A subgroup III in Africa, 1995. Bull World Health Organ. 1996;74:181–7. 8706234 PMC2486908

[7] Cunin P, Fonkoua MC, Kollo B, Bedifeh BA, Bayanak P, Martin PM, *Neisseria meningitidis* outside meningitis belt in southwest Cameroon. Emerg Infect Dis. 2003;9:1351–3. 14626225 10.3201/eid0910.030170 PMC3033097

